# Inhibition of GPR91 Reduces Inflammatory Mediators Involved in Active Labor in Myometrium

**DOI:** 10.1155/2020/6454282

**Published:** 2020-04-15

**Authors:** Ratana Lim, Martha Lappas

**Affiliations:** ^1^Obstetrics, Nutrition and Endocrinology Group, Department of Obstetrics and Gynaecology, University of Melbourne, Victoria, Australia; ^2^Mercy Perinatal Research Centre, Mercy Hospital for Women, Heidelberg, Victoria, Australia

## Abstract

**Results:**

GPR91 mRNA expression was significantly higher in myometrium from women during term spontaneous labor compared to no labor. Likewise, in mice, GPR91 mRNA expression was significantly upregulated in myometrium during inflammation-induced preterm labor compared to preterm no labor. In myometrial cells, IL1B and TNF significantly increased GPR91 mRNA expression. Knockdown of GPR91 by siRNA in myometrial cells significantly suppressed the secretion and/or expression of IL1B- and TNF-induced proinflammatory cytokines (GM-CSF, IL1A, IL1B, and IL6) and chemokines (CXCL8 and CCL2), myometrial contractility (expression of the contraction-associated proteins PTGFR and CX43, secretion of the uterotonic PGF_2*α*_, and *in situ* collagen gel contraction), and the transcription factor NF-*κ*B.

**Conclusion:**

Our findings demonstrate that GPR91 is involved in the genesis of proinflammatory and prolabor mediators induced by IL1B or TNF and collectively suggest that GPR91 may contribute to augmentation of the labor processes.

## 1. Introduction

G-protein-coupled receptors (GPCRs) are the largest family of membrane receptors involved in signal transduction [1], and as such, they can participate in many physiological and pathological processes. This makes GPCRs an attractive drug target for treating human diseases. Classification into different families is based on sequence homology and to the types of ligands they can bind. Some GPCRs have been identified as important regulators of inflammation; both exacerbating inflammation and promoting its resolution [2]. Notably, several GPCRs have been shown to play a role in myometrial activation. These include the prostaglandin F receptor PTGFR [3, 4] and the lactate receptor GPR81 [5]. GPR91 has recently been shown to be involved in the pathogenesis of several physiological and pathological conditions associated with inflammation including rheumatoid arthritis [6] and obesity and diabetes [7, 8]. The role of GPR91 in myometrial activation, however, is not known.

GPR91 is considered to be a class A orphan GPCR; however, under some circumstances, the Krebs cycle intermediate succinate can serve as a ligand for GPR91 [9], and thus, it is also known as succinate receptor 1, SUCNR1. Succinate, however, exerts many non-receptor-mediated effects [10] and thus is not a reliable ligand to define the exact biological functions of GPR91. Nevertheless, targeted studies have shown that activation of GPR91 initiates a complex signal transduction cascade that leads to the upregulation of inflammatory markers. For example, there is increased expression of prostaglandin-endoperoxide synthase 2 (PTGS2) in mice overexpressing GPR91 [11] while knockdown of GPR91 inhibited PTGS2 expression and levels of prostaglandin E_2_ (PGE_2_) in diabetic rats [12] and attenuated pathological alterations in retinal vessels in a rat model of oxygen-induced retinopathy [13]. Concordantly, GPR91 has been shown to be dysregulated in many inflammatory pathologies. GPR91 is involved in the development of hypoxic retinal vascular diseases [14] while GPR91 knockout mice developed reduced arthritic disease [15].

There are no studies on GPR91 in myometrium, or its role in regulating inflammatory mediators involved in active labor. Thus, the first aim of this study was to characterise the expression of GPR91 in myometrium from laboring and nonlaboring women, in a mouse model of inflammation-induced preterm birth and in the presence of proinflammatory cytokines. On the basis of positive findings, the second aim was to determine the effect of siRNA knockdown of GPR91 on prolabor mediators in human primary myometrial cells.

## 2. Materials and Methods

### 2.1. Tissue Collection

Approval for this study was obtained from the Mercy Hospital for Women's Research and Ethics Committee, and written informed consent was obtained from all participating subjects. Women were invited to provide samples on the day of admission for surgery. Myometrium was obtained from the upper margin of the lower uterine segment incision during Caesarean section at term (37-41 weeks of gestation). Tissues were processed within 15 mins of delivery as we have previously described [16]. For the cell culture experiments, myometrium was obtained from women who delivered healthy, singleton infants at term undergoing elective Caesarean section in the absence of labor. For the expression studies, myometrium was obtained from women at term Caesarean section (i) in the absence of labor or (ii) during active spontaneous labor (*n* = 11 patients per group) as previously described [17]. In the absence of labor, indications for Caesarean section were breech presentation and/or previous Caesarean section. In the laboring group, indications for Caesarean section were for fetal malpresentation, fetal distress, and delayed or failure to progress. Myometrium was obtained from women who delivered healthy, singleton infants. Exclusions were women with obesity, diabetes, asthma, polycystic ovary syndrome, preeclampsia and multiple pregnancies, and fetuses with chromosomal abnormalities. Clinical details of the patients used in this study are described in Table 1.

### 2.2. Primary Myometrial Cell Culture

Primary human myometrial cells were used to determine the effect of proinflammatory cytokines on GPR91 expression. Myometrial cells, isolated as previously described [16], were incubated in the absence or presence of 1 ng/ml IL1B (PeproTech; Rocky Hill, NJ, USA) or 10 ng/ml TNF (PeproTech; Rocky Hill, NJ, USA) for 5 or 20 h after which cells were collected and stored at −80°C until assayed for GPR91 mRNA expression by RT-qPCR as detailed below. The concentrations of IL1B and TNF are based on previous studies in myometrial cells [18]. Experiments were performed from myometrium obtained from 5 patients.

To investigate if GPR91 regulates mediators involved in active labor, we performed siRNA knockdown experiments in primary myometrial cells. Myometrial cells (~60% confluency) were transfected with 50 nM GPR91 siRNA (siGPR91) or negative control siRNA (siCONT) (Ambion; Thermo Fisher Scientific; Scoresby, Vic, Australia) using Lipofectamine 3000 (Life Technologies; Mulgrave, Victoria, Australia) per the manufacturer's guidelines. After 48 h, cells were treated with or without 1 ng/ml IL1B or 10 ng/ml TNF. After 20 h incubation, cells and media were collected and stored at −80°C until analysed as detailed below. A MTT proliferation assay was used to assess cell viability as we have previously described [19]. Experiments were performed from myometrium obtained from 8 patients.

### 2.3. Myometrial Cell Gel Contraction Assay

To determine the effect of siGPR91 on myometrial cell contractility, gel contraction assays were performed as previously described [20]. Briefly, siCONT- or siGPR91-transfected primary myometrial cells were resuspended in 0.25 ml DMEM/F12 (containing 10% FBS) containing collagen (3 mg/ml collagen I from rat protein solution; Gibco™) and 1 *μ*l 1 M NaOH. The mixture was transferred to 48-well tissue culture plates and incubated at 37°C for 1 h to allow polymerization. Gels were then treated with or without 10 ng/ml TNF for 36 h. The area of the gel was determined using Image Lab software (Bio-Rad Laboratories, Hercules, CA, USA). Cell contraction was performed using myometrium obtained from six patients.

### 2.4. NF-*κ*B RELA Luciferase Assay

To determine the effect of siGPR91 on NF-*κ*B activation in primary myometrial cells, a luciferase assay was performed as previously described [21]. Briefly, primary myometrial cells were transfected with 0.1 *μ*g NF-*κ*B RELA reporter construct (Qiagen; Chadstone Centre, Vic, Australia) with FuGENE HD transfection reagent (Promega; Alexandria NSW, Australia) for 48 h. Thereafter, the cells were treated with or without 1 ng/ml IL1B or 10 ng/ml TNF for 20 h. The ratio of the firefly luciferase level to the Renilla luciferase level was determined, and the results are expressed as a ratio of normalised luciferase activity. Experiments were performed from myometrium obtained from six patients.

### 2.5. Mouse Studies

To determine GPR91 expression in myometrium during preterm labor, we used a mouse model of inflammation-induced preterm labor. Mouse studies, conducted with approval from the Austin Health's Animal Ethics Committee, were performed as we have previously described [20]. Briefly, eight-week-old timed-pregnant C57BL/6 female mice (purchased from WEHI, Melbourne, Australia) were injected intraperitoneally with LPS (serotype O26:B6; 15 *μ*g in 50 *μ*l of PBS; Sigma) or sterile PBS (vehicle control) on gestational day 15.5. As we have previously reported [20], this results in a high rate of preterm delivery (18-22 h post-LPS treatment) and does not cause maternal mortality. None of the vehicle-injected mice went into labor. The mice were killed on the birth of one pup, and time-matched controls were killed directly afterward. Myometrial tissue was washed in PBS, flash frozen, and stored at -80°C until further analysis by RT-qPCR as detailed below.

### 2.6. RNA Extraction and RT-qPCR

RNA extractions, cDNA synthesis, and RT-qPCR were performed as previously described [22] using 100 nM of predesigned and validated QuantiTect primers (primer sequences not available) (Qiagen; Chadstone Centre, Vic, Australia). Target gene Ct values were normalised to the average YWHAZ and succinate dehydrogenase (SDHA) Ct values of the same cDNA sample and fold differences determined using the comparative Ct method. Of note, there was no effect of treatment on YWHAZ or SDHA Ct values.

### 2.7. Enzyme Immunoassays

The levels of IL6 and CXCL8 in the incubation media were measured using sandwich ELISA from Invitrogen per the manufacturer's instructions. The levels of CCL2, CXCL1, and sICAM1 in the incubation media were measured by sandwich ELISA from R&D Systems (Minneapolis, MN, USA) per the manufacturer's instructions. The release of PGF_2*α*_ into the incubation medium was assayed using a commercially available competitive enzyme immunoassay kit per the manufacturer's specifications (Cayman Chemical Company; Ann Arbor, MI, USA). Interassay and intra-assay coefficients of variation for all assays were less than 10%.

### 2.8. Statistical Analysis

All statistical analyses were undertaken using GraphPad Prism (GraphPad Software, La Jolla, CA, USA). Normality of the data was assessed using the Shapiro-Wilk test. For two sample comparisons, an unpaired Student's *t*-test was used to assess statistical significance between normally distributed data; otherwise, the Mann-Whitney *U* test was used. For all other comparisons, data were analysed by a repeated measures one-way ANOVA (with LSD post hoc testing to discriminate among the means); nonnormally distributed data were logarithmically transformed before analysis. Statistical significance was ascribed to a *P* value ≤ 0.05. Data is expressed as mean ± SEM.

## 3. Results

### 3.1. Characterisation of GPR91 Expression in Myometrium before and after Labor

**Figure 1:**
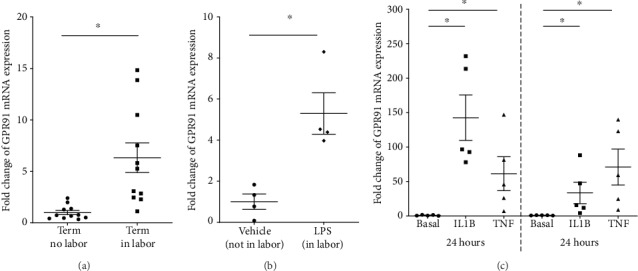
Expression of GPR91 in myometrium. (a) Human myometrium was obtained from women at term Caesarean section in the absence of labor (term no labor, *n* = 11 patients) or from women at term Caesarean section during labor (term in labor, *n* = 11 patients). GPR91 mRNA expression was analysed by RT-qPCR, and the fold change was calculated relative to the term no labor group. Individual data points represent different patients, and the horizontal line represents the mean ± SEM of each group. ^∗^*P* ≤ 0.05, Mann-Whitney *U* test. (b) Myometrium was obtained from mice during LPS-induced labor at 15.5 dpc and time-matched vehicle-injected mice (*n* = 4 mice/group). GPR91 mRNA expression was analysed by RT-qPCR, and the fold change was calculated relative to the vehicle group. Individual data points represent different mice, and the horizontal line represents the mean ± SEM of each group. ^∗^*P* ≤ 0.05, unpaired *t*-test. (c) Human primary myometrial cells were incubated in the absence or presence of 10 ng/ml IL1B or 10 ng/ml TNF for 5 h or 20 h (*n* = 5 patients). GPR91 mRNA expression was analysed by RT-qPCR, and fold change was calculated relative to basal. Individual data points represent 5 independent experiments, and the horizontal line represents the mean ± SEM of each group. ^∗^*P* ≤ 0.05, repeated measures one-way ANOVA.

There was a significant increase in GPR91 mRNA expression in myometrium obtained at term Caesarean section during spontaneous labor onset (term, in labor), compared to nonlaboring tissue (term, no labor) (Figure 1(a)). A mouse model was utilised to determine the effect of preterm labor, where day 15.5 dpc mice were injected with LPS and myometrium was collected at delivery of first pup.  Figure 1(b) demonstrates that GPR91 mRNA expression was significantly higher, a 5-fold increase, in mouse myometrium with preterm (LPS) labor. Next, myometrial cells were treated with proinflammatory cytokines IL1B and TNF, which are known labor mediators.  Figure 1(c) shows that treatment with both IL1B and TNF significantly increased GPR91 mRNA expression at both 5 and 24 h time points; notably, the increase with IL1B treatment was ~3-fold higher after 5 h treatment than at 24 h.

### 3.2. Effect of GPR91 siRNA Knockdown on Proinflammatory Cytokines and Chemokines in Human Primary Myometrial Cells *In Vitro*

To determine the specific role of GPR91 in the regulation of prolabor mediators, gene knockdown was achieved using siRNA (siGPR91) in human primary myometrial cells. There was an 82% decrease in GPR91 mRNA expression in myometrial cells transfected with siGPR91. A MTT cell viability assay showed no change in absorbance between cells transfected with siCONT or siGPR91 (data not shown). For subsequent experiments, transfected cells were treated with IL1B or TNF as models of inflammation associated with labor. For these experiments, we chose a 20 h time point as 5 h incubation is not enough time to induce cytokine, chemokine, and prostaglandin release.

**Figure 2:**
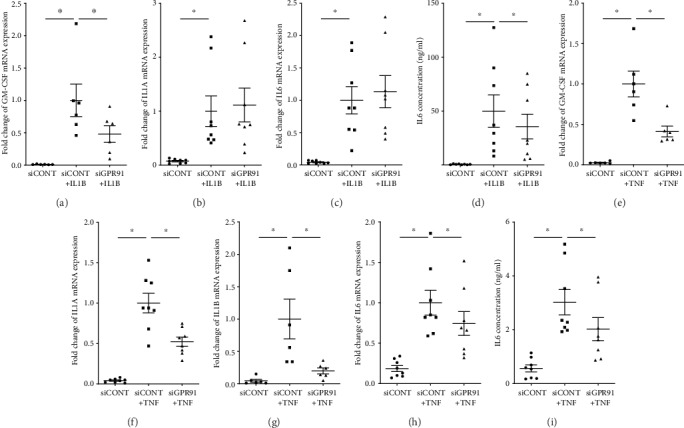
Effect of GPR91 siRNA knockdown on proinflammatory cytokines. Human primary myometrial cells were transfected with 50 nM siCONT or 50 nM siGPR91 and then treated with 1 ng/ml IL1B or 10 ng/ml TNF (*n* = 6‐8 patients). (a–c, e–h) GM-CSF, IL1A, IL1B, and IL6 mRNA expression was analysed by RT-qPCR. (d, i) The concentration of IL6 in the incubation medium was assayed by ELISA. For all data, the fold change was calculated relative to siCONT+IL1B- or siCONT+TNF-transfected cells. Individual data points represent 6-8 independent experiments, and the horizontal line represents the mean ± SEM of each group. ^∗^*P* ≤ 0.05, repeated measures one-way ANOVA.

Figure 2 demonstrates the effect of siGPR91 on the expression of proinflammatory cytokines. Treatment of siCONT-transfected cells with IL1B significantly increased GM-CSF, IL1A, and IL6 mRNA expression and release of IL6 (Figures 2(a)–2(d)). There was no effect of siGPR91 on IL1B-induced IL1A or IL6 mRNA expression (Figures 2(a) and 2(b)); however, there was a significant decrease in IL1B-induced GM-CSF mRNA expression (Figure 2(a)) and IL6 release in siGPR91-transfected cells (Figure 2(d)). Treatment of siCONT-transfected cells with TNF significantly increased GM-CSF, IL1A, IL1B, and IL6 mRNA expression (Figures 2(e)–2(h)) and release of IL6 (Figure 2(i)). This increase was significantly lower in siGPR91-transfected cells. The levels of IL1A, IL1B, and GM-CSF in the incubation media were below the sensitivity of the assay and thus not assessed.

**Figure 3:**
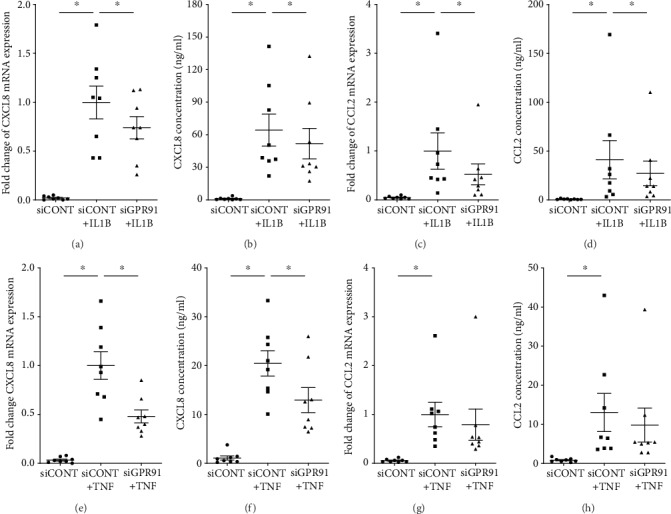
Effect of GPR91 siRNA knockdown on proinflammatory chemokines. Human primary myometrial cells were transfected with 50 nM siCONT or 50 nM siGPR91 and then treated with 1 ng/ml IL1B or 10 ng/ml TNF (*n* = 8 patients). (a, c, e, and g) CXCL8 and CCL2 mRNA expression was analysed by RT-qPCR. (b, d, f, and h) The concentration of CXCL8 and CCL2 in the incubation medium was assayed by ELISA. For all data, the fold change was calculated relative to siCONT+IL1B- or siCONT+TNF-transfected cells. Individual data points represent 6 independent experiments, and the horizontal line represents the mean ± SEM of each group. ^∗^*P* ≤ 0.05, repeated measures one-way ANOVA.

The effect of siGPR91 on the expression of proinflammatory chemokines is demonstrated in Figure 3. There was an expected increase in CXCL8 and CCL2 mRNA expression and release in siCONT-transfected cells treated with IL1B (Figures 3(a)–3(d)) and TNF (Figures 3(e)–3(h)). In siGPR91-transfected cells, there was a significant decrease in CXCL8 mRNA expression and release in the presence of IL1B (Figures 3(a) and 3(b)) and TNF (Figures 3(e) and 3(f)). There was a significant decrease in IL1B-induced CCL2 mRNA expression and secretion (Figures 3(c) and 3(d)), but there was no effect of siGPR91 on TNF-induced CCL2 mRNA expression and secretion (Figures 3(g) and 3(h)).

### 3.3. Effect of GPR91 siRNA Knockdown on Myometrial Cell Contractility *In Vitro*

**Figure 4:**
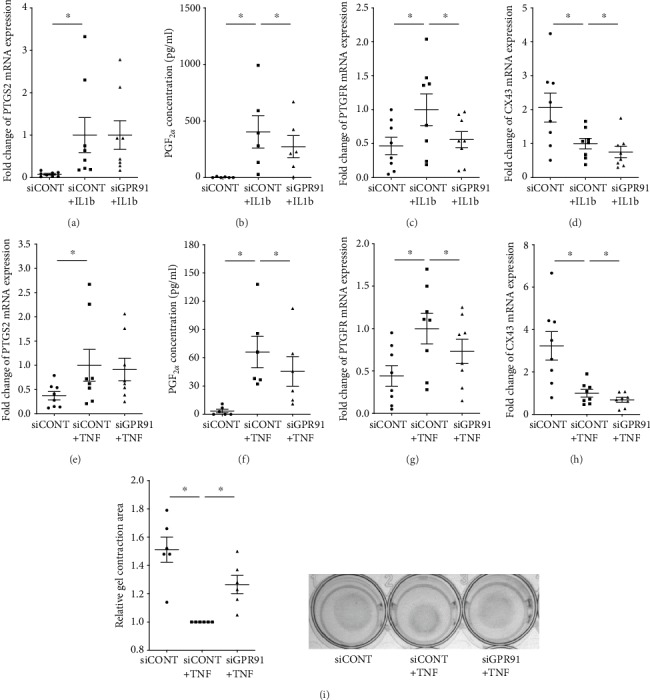
Effect of GPR91 siRNA knockdown on myometrial cell contractility. (a–f) Human primary myometrial cells were transfected with 50 nM siCONT or 50 nM siGPR91 and then treated with (a–d) 1 ng/ml IL1B or (e–h) 10 ng/ml TNF (*n* = 8 patients). (a, c, d, e, g, and h) PTGS2, PTGFR, and CX43 mRNA expression was analysed by RT-qPCR. (b, f) The concentration of PGF_2*α*_ in the incubation medium was assayed by ELISA. Individual data points represent 6 independent experiments, and the horizontal line represents the mean ± SEM of each group. ^∗^*P* ≤ 0.05, repeated measures one-way ANOVA. (i) Cell contraction assays were performed using collagen gels made from human primary myometrial cells transfected with 50 nM siCONT or 50 nM siGPR91 for 48 h (*n* = 6 patients). The collagen gels were then treated with or without 10 ng/ml TNF for 36 h, and the area of gel was determined. Representative gel contraction image from 1 patient is also shown. For all data, the fold change was calculated relative to siCONT+IL1B- or siCONT+TNF-transfected cells. Individual data points represent 6 independent experiments, and the horizontal line represents the mean ± SEM of each group. ^∗^*P* ≤ 0.05, repeated measures one-way ANOVA.

The effect of siGPR91 on mediators involved in uterine contractility is depicted in Figure 4. Uterine contractions are induced through increased expression of contraction-associated proteins such as PTGS2, which leads to increased production of uterotonic prostaglandins such as PGF_2*α*_. There was no effect of siGPR91 on the expression of PTGS2 mRNA in the presence of IL1B (Figure 4(a)) or TNF (Figure 4(e)). There was a significant decrease in IL1B- and TNF-induced PGF_2*α*_ secretion (Figures 4(b) and 4(f)) and PTGFR mRNA expression (Figures 4(c) and 4(g)) in siGPR91-transfected cells. There was a significant decrease in CX43 mRNA expression in siCONT-transfected cells treated with IL1B (Figure 4(d)) and TNF (Figure 4(h)). In siGPR91-transfected cells, there was a significant decrease in IL1B- and TNF-induced CX43 mRNA expression (Figures 4(d) and 4(h)). No change in OXTR mRNA expression in these cells (data not shown). Collagen gel contraction assays were performed to assess whether siGPR91 could affect myometrial cell contraction. Human myometrial cells stimulated with TNF induced contraction of the collagen gels (as shown by reduced gel area) after 24 h (Figure 4(i)). This TNF-induced contraction was significantly diminished in siGPR91-transfected cells.

### 3.4. Effect of GPR91 siRNA Knockdown on RELA Transcriptional Activity NF-*κ*B Activation in Human Primary Myometrial Cells *In Vitro*

**Figure 5:**
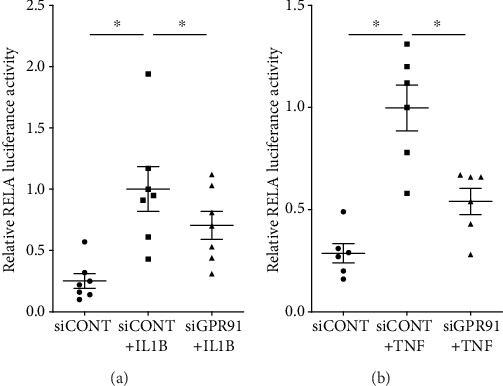
Effect of GPR91 siRNA knockdown on RELA transcriptional activity. Human myometrial cells were transfected with 0.75 ng RELA reporter construct. After 6 h, cells were transfected with 50 nM siCONT or 50 nM siGPR91 for 48 h, then treated with (a) 1 ng/ml IL1B (*n* = 7 patients) or (b) 10 ng/ml TNF for an additional 20 h (*n* = 6 patients). RELA promoter activity (normalised to Renilla) is expressed as a ratio of luciferase activity. For all data, the fold change was calculated relative to siCONT+IL1B- or siCONT+TNF-transfected cells. Individual data points represent independent experiments, and the horizontal line represents the mean ± SEM of each group. ^∗^*P* ≤ 0.05, repeated measures one-way ANOVA.

We found that proinflammatory cytokines IL1B and TNF induce GPR91 mRNA expression in myometrial cells and that GPR91 inhibition, using siRNA, decreased the expression of inflammation-induced cytokines, chemokines, and contraction-associated proteins. We hypothesize that GPR91 gene silencing inhibits NF-*κ*B activation, to reduce the production of these prolabor mediators. To assess this, we transfected myometrial cells with a NF-*κ*B RELA luciferase construct and determined its activity in response to siGPR91. In siCONT-transfected cells, there was a significant increase in RELA transcriptional activity (i.e., NF-*κ*B activation) when treated with IL1B (Figure 5(a)) and with TNF (Figure 5(b)) compared to basal. In siGPR91-transfected cells, there was a significant decrease in both IL1B-induced (Figure 5(a)) and TNF-induced (Figure 5(b)) RELA transcriptional activity.

## 4. Discussion

For the first time, we report that GPR91 is upregulated in laboring myometrium and in response to the proinflammatory mediators IL1B and TNF. Further, we have identified GPR91 as a regulator of inflammation-induced proinflammatory and prolabor events in myometrium, namely, proinflammatory cytokines, chemokines, and mediators involved in myometrial contractility. Abnormal GPR91 expression may thus create a positive autocrine loop, which results in the continuous stimulation of inflammation, thereby augmenting the labor processes.

We demonstrate that the expression of GPR91 is significantly increased in term myometrium at labor compared with nonlaboring tissues. Labor-associated changes in GPR91 expression were only assessed in myometrial samples obtained from women at term gestation. While it is important to also assess if the increase in GPR91 expression is also evident during preterm labor, these samples are extremely difficult to obtain and rarely free of confounding factors. To overcome this limitation, we used a mouse model of preterm labor induced by LPS. Myometrial samples were obtained from mice during labor and from gestation-matched mice in the absence of labor. We found that LPS induced a significant upregulation of GPR91 mRNA expression in myometrium when compared with vehicle-injected controls. It is important to note that these were taken from mice during labor; thus, whether GPR91 is involved in the transition of the myometrium from a quiescent into a contractile state in preparation for labor is not known. Temporal-associated changes in GPR91 expression would be able to address this. Obtaining myometrial biopsies from early/mid pregnancy from women is extremely difficult. Myometrium, however, can be obtained to examine changes in GPR91 from mice before the activation of labor cascade.

Whether increased GPR91 expression is a cause or consequence of labor is not known. Laboring myometrium, however, is characterised by increased expression of lymphocytes [23] which are a rich source of proinflammatory cytokines such as IL1B and TNF. IL1B and TNF are acute phase proteins that constitute an initial proinflammatory stimulus to augment the inflammatory response of labor [24, 25]. Thus, to determine the mechanisms responsible for this increase in GPR91 during labor, we incubated myometrial cells with IL1B and TNF and found a robust and highly significant upregulation of GPR91 mRNA expression at 5 h post treatment, which persisted at 24 h post treatment. Collectively, these findings suggest that the increase in GPR91 in laboring myometrium may be a consequence of the labor process where it may be involved in propagating the inflammatory response of labor.

We then performed functional studies to determine if GPR91 participates in IL1B- or TNF-induced proinflammatory and prolabor mediators. We found that GPR91 silencing was associated with a downregulation of IL1B- or TNF-induced mRNA expression and protein secretion of proinflammatory cytokines (GM-CSF, IL1A, IL1B, and IL6) and chemokines (CXCL8 and CCL2). Interestingly, siGPR91 exerted differential effects in response to IL1B or TNF. For example, siGPR91 was more efficient in abrogating proinflammatory cytokines induced by TNF compared to IL1B. This may be due to IL1B and TNF using different signaling pathways and recruit distinct adaptor molecules to regulate target genes; IL1B is known to propagate inflammation by stimulating the MyD88 pathway [24], while TNF acts through TRADD signaling [25]. In summary, siRNA knockdown of GPR91 reduces the effects inflammatory mediators have in promoting mediators involved in myometrial activation and suggests that GPR91 is part of the process by which inflammation induces parturition.

Our results also demonstrate that GPR91 can induce myometrial contractility *in vitro*. This was demonstrated by incubating myometrial cells deficient in GPR91 with collagen and determining their response to TNF. Of note, we found that TNF-induced contraction (i.e., shrinkage) of collagen gel matrices was suppressed in the myometrial cells transfected with GPR91. Our results indicate that elevated expression of GPR91 in laboring myometrium may augment uterine contractions during labor. This is further reinforced by the regulatory effect of GPR91 on the expression of contraction-associated proteins PTGFR and CX43 and the secretion of the contractile agonist PGF_2*α*_. Of note, there was no effect of siGPR91 on PTGS2 expression suggesting that GPR91 may regulate enzyme downstream of PTGS2 to regulate the synthesis of PGF_2*α*_. In addition to its role in regulating myometrial inflammation [26], PGF_2*α*_, which signals via the PTGFR, has a pivotal role in inducing contractions of the uterus by increasing intracellular calcium levels [27]. Further evidence for a role of GPR91 in regulating myometrial contractility is demonstrated by the effect of siGPR91 on the expression of the proinflammatory cytokines and the chemokines known to be involved in uterine contractility. IL1B enhances contractility by promoting a calcium influx into uterine smooth muscle cells [28], while the chemokine CXCL8 has been shown to potentiate the effect of IL1B on uterine contractions [29]. Taken together, we demonstrate, for the first time, that GPR91 is required for myometrial contractility and that GPR91 regulates inflammatory signals that activate the myometrium to a contractile state.

We finally interrogated whether GPR91 elicits its actions via the proinflammatory and prolabor transcription factor NFKB1. There is compelling evidence to demonstrate a vital role for NFKB1 in parturition [30, 31]. Notably, in human primary myometrial cells, we have shown that NFKB1 regulates IL1B- and TNF-induced expression and secretion of proinflammatory and prolabor mediators [24, 25]. In this study, GPR91 siRNA knockdown in primary myometrial cells was associated with a suppression of RELA transcriptional activity in response to IL1B or TNF. Therefore, for these results, we can surmise that IL1B and TNF may regulate prolabor mediators via GPR91 activation of NFKB1.

We used *in vitro* siRNA studies to demonstrate a role for GPR91 in regulating the inflammatory response in myometrial cells. Whether GPR91 also controls the inflammatory cascade in myometrium during infection is not known and is an avenue for further research. In addition, based on our previous studies [18], we only chose one dose of IL1B and TNF. These doses have been shown to induce expression and secretion of proinflammatory and prolabor mediators in myometrial cells [18]. A dose response study would be able to show the physiologic versus pathologic impact of proinflammatory cytokines on GPR91 expression and on prolabor mediators in response to GPR91 siRNA knockdown.

In conclusion, using both human clinical samples and a mouse model of inflammation-induced preterm labor, we have established that GPR91 is upregulated with labor and by proinflammatory insults. We also established a new role for the GPCR, GPR91, in regulating inflammatory mediators that play key roles in the physiological characteristics of labor, namely, proinflammatory cytokines, chemokines, and contraction-associated proteins. Animal studies are required to determine if GPR91 antagonists can prevent intrauterine inflammation and delay preterm labor.

## Figures and Tables

**Table 1:** Clinical characteristics of the patients.

	Term no labor *n* = 11 patients	Term in labor *n* = 11 patients
Maternal age (years)	31.9 (1.3)	31.6 (1.5)
Prepregnancy maternal BMI	22.5 (0.9)	23.5 (1.0)
Gravida	2.6 (0.2)	2.6 (0.5)
Parity	2.2 (0.2)	2.4 (0.4)
Gestational age (weeks)	38.9 (0.2)	39.2 (0.4)
Fetal gender	5 female; 6 male	5 female; 6 male
Birth weight (g)	3291 (155)	3543 (82)
Labor		
No labor	100%	0%
Spontaneous	0%	100%
Duration of labor (min)	N/A	626 (75)
Membrane rupture		
SROM	0%	36.4%
PROM	0%	0%
ARM	100%	63.6%

Values represent mean (±SEM) unless otherwise specified.

## Data Availability

The data used to support the findings of this study are included within the article.
